# Characterization and Functional Analysis of Calmodulin and Calmodulin-Like Genes in *Fragaria vesca*

**DOI:** 10.3389/fpls.2016.01820

**Published:** 2016-12-01

**Authors:** Kai Zhang, Dingyi Yue, Wei Wei, Yang Hu, Jiayue Feng, Zhirong Zou

**Affiliations:** ^1^Key Laboratory of Protected Horticultural Engineering in Northwest, Ministry of AgricultureYangling, China; ^2^State Key Laboratory of Crop Stress Biology for Arid Areas, Northwest A&F UniversityYangling, China

**Keywords:** strawberry (*Fragaria vesca.*), bioinformatics analysis, expression profile, subcellular localization, transient assay

## Abstract

Calcium is a universal messenger that is involved in the modulation of diverse developmental and adaptive processes in response to various stimuli. Calmodulin (CaM) and calmodulin-like (CML) proteins are major calcium sensors in all eukaryotes, and they have been extensively investigated for many years in plants and animals. However, little is known about CaMs and CMLs in woodland strawberry (*Fragaria vesca*). In this study, we performed a genome-wide analysis of the strawberry genome and identified 4 *CaM* and 36 *CML* genes. Bioinformatics analyses, including gene structure, phylogenetic tree, synteny and three-dimensional model assessments, revealed the conservation and divergence of *FvCaMs* and *FvCMLs*, thus providing insight regarding their functions. In addition, the transcript abundance of four *FvCaM* genes and the four most related *FvCML* genes were examined in different tissues and in response to multiple stress and hormone treatments. Moreover, we investigated the subcellular localization of several FvCaMs and FvCMLs, revealing their potential interactions based on the localizations and potential functions. Furthermore, overexpression of five *FvCaM* and *FvCML* genes could not induce a hypersensitive response, but four of the five genes could increase resistance to *Agrobacterium tumefaciens* in *Nicotiana benthamiana* leaves. This study provides evidence for the biological roles of *FvCaM* and *CML* genes, and the results lay the foundation for future functional studies of these genes.

## Introduction

Ca^2+^, which functions as a universal second messenger, plays vital roles in the developmental processes of plants and in response to various environmental stimuli (Trewavas and Malho, 1998; Berridge et al., 2000). Plants have evolved a diversity of unique proteins that bind to Ca^2+^, most commonly via the evolutionarily conserved EF-hand motif (Clapham, 2007). This conserved motif has been extensively studied (Kawasaki et al., 1998; Gifford et al., 2007). More than 250 proteins containing EF-hands have been found in the *Arabidopsis* genome (Day et al., 2002). CaM, as well as CML, one of the most well described Ca^2+^ sensors, is composed of EF-hands and is essential for calcium signal transduction in plants.

Calmodulins are highly conserved among eukaryotic proteins, exhibiting almost 90% identity in humans and plants. In contrast, CMLs are restricted to plants (DeFalco et al., 2010). CaMs are encoded by only a few *CaM* genes in animal genomes (e.g., three in humans), whereas plant genomes carry a greater number of *CaM* genes and multiple loci containing identical *CaM* genes (DeFalco et al., 2010). In plant genomes, the *CaM/CML* gene families are represented by seven *CaM* genes, including two sets of isoforms encoding identical proteins (e.g., *CaM1* and *CaM4*; *CaM2*, *CaM3*, and *CaM5*), 50 *CML* genes in *Arabidopsis* (McCormack and Braam, 2003), and five *CaM* genes and 32 *CML* genes in rice (Boonburapong and Buaboocha, 2007).

The conservation of CaMs reflects the strict restrictions on the structure of the proteins. A typical CaM contains four EF-hand motifs (Snedden and Fromm, 2001), but for CMLs, this number ranges from one to six, and some of the motifs are degraded (McCormack and Braam, 2003; Boonburapong and Buaboocha, 2007). Structurally, the EF-hand motif is composed of two helices, the E helix and the F helix, flanking a Ca^2+^-binding loop in a structure that resembles a hand (McCormack et al., 2005). The crystal structure of CaMs has revealed that these proteins comprise two globular domains (the N-lobe and the C-lobe) (Babu et al., 1984), each containing a pair of EF-hands with affinity for Ca^2+^ (Tidow and Nissen, 2013). The binding of Ca^2+^ to CaMs induces a conformational change that results in exposure of the hydrophobic surface, which then interacts with downstream CaM-binding proteins (CBPs) (Kursula, 2014).

As essential Ca^2+^ sensors, CaMs are involved in a series of developmental processes, especially in stress-induced signaling pathways. Accumulating evidence indicates that gene over-expression or loss of function in plants carrying mutations in *CaM* and *CML* genes strongly affects the immune system. For example, down-regulation of the pathogen-induced *CaM* genes, *NtCaM1* and *NtCaM13*, was found to differentially impact disease resistance in tobacco (Takabatake et al., 2007). The loss-of-function *cml9* mutation in *Arabidopsis* was shown to enhance susceptibility to pathogens, and over-expression of *AtCML9* reduced susceptibility to virulent strains of *Pst* (Magnan et al., 2008). In addition to biotic stress, CaMs and CMLs are also involved in abiotic stress responses. *GmCaM4*, a salt-stress induced gene, has been reported to mediate Ca^2+^ signaling and the downstream transcription factor (TF), *MYB2* (Rao et al., 2014). In addition, a *CaM3* knockout *Arabidopsis* mutant displayed impaired thermotolerance, whereas overexpression of *AtCaM3* increased plant thermotolerance, via interactions with heat stress transcription factors (HSFs) (Zhang et al., 2009). Moreover, a series of studies have demonstrated the roles of CaMs and CMLs in hormone signaling (Kaplan et al., 2006; Du et al., 2009).

To date, no systematic research has been conducted to investigate *CaM* and *CML* genes in strawberry. In the present study, we performed a genome-wide analysis and identified 4 *CaM* genes and 36 *CML* genes in the woodland strawberry genome (*Fragaria vesca*). The gene structure analysis suggested that *FvCaMs* possess a conserved motif organization, while *FvCMLs* possess complex structures in terms of both motif organization and exon–intron structure. Phylogenetic tree and synteny analyses provided insight regarding the evolution and function of *FvCaM* and *FvCML* genes. Using AtCaM7 as template, we predicted the three-dimensional structure of FvCaM1, thus facilitating our understanding of the mechanisms of FvCAMs. In addition, we assessed the transcript abundance in different tissues and in response to multiple stress and hormone treatments. Furthermore, we investigated the subcellular localization of several FvCaMs and CMLs in *Arabidopsis* mesophyll protoplasts under normal condition or in response to diverse treatments. We also performed transient assays to explore the biological roles of FvCaMs and FvCMLs in *Nicotiana benthamiana* leaves. Our results provide a subset of FvCaM and CML genes that could be targeted in future engineering strategies to modify both pathogen resistance and stress tolerance in strawberry.

## Materials and Methods

### Identification of *CaM* and *CML* Genes in the Strawberry Genome

To identify *CaM* and *CML* homologs in the strawberry genome (*F. vesca*), we performed a Blast-P search against the RefSeq protein database of *F. veca* in NCBI with the default parameters. The amino acid sequence of *AtCaM1* gene was chosen as the query. After then, we manually chose the proteins with the sequence identity of more than 85 and 16% as putative CaMs and CMLs, respectively. All of the putative candidates were manually verified using the online software InterProScan^1^) and Pfam^2^ to check their completeness and to confirm the presence of the EF-hand domain. The phylogenetic relationships of the candidates were determined to confirm that they were *FvCaM* or *FvCML* members. The corresponding nucleotide sequences as well as information regarding each gene were obtained from NCBI. The sequences of *Arabidopsis* and rice *CaM* and *CML* genes were obtained from the *Arabidopsis* Information Resource (TAIR^3^) and rice genome database in TIGR^4^, respectively.

### Evolutionary and Structural Analyses

The amino acid sequences of CaMs and CMLs from *Arabidopsis*, rice and strawberry were aligned using ClustalX with default settings. The phylogenetic tree was constructed with MEGA5.0 using the neighbor-joining method and a bootstrap setting of up to 1000 replicates. The organization of the FvCaM and FvCML motifs was derived using the online tool MEME^5^ and visualized by DOG 2.0^6^. Analysis of the exon–intron structure was conducted using GSDS 2.0^7^ by aligning the cDNA sequences with their corresponding genomic DNA sequences. Synteny blocks between *Arabidopsis* and strawberry genomes were obtained from the Plant Genome Duplication Database^8^. All of the identified relevant *CaM* and *CML* genes were visualized using Circos (Krzywinski et al., 2009) and Adobe Photoshop CS6.

The FvCaMs amino acid sequences were aligned with AtCaM7 using ClustalX version 2.0.12 and embellished using GeneDoc. The three-dimensional structure was predicted and visualized by Swiss-Model^9^ using *Arabidopsis* AtCaM7 as the template (Tidow et al., 2012). The structural details and annotations are presented in reference to a previously published paper (McCormack et al., 2005).

### Plant Materials and Treatments

The Chinese woodland strawberry, *F. vesca* accession Heilongjiang-3, was grown in the greenhouse at Northwest A&F University in China at a temperature of 18–23°C, relative humidity of 60–80%, and without supplemental lighting. Three-month-old strawberry seedlings, at which time the sixth leaf was fully expanded, were selected for the treatments. *Arabidopsis thaliana* ecotype Col-0 plants were grown at 22°C with a relative humidity of 75% and under short-day (8 h of light at 125 μmol⋅m^-2^⋅s^-1^, 16 h of dark) conditions for 4–5 weeks before transformation.

The PM treatment experiment was conducted by touching the adaxial epidermis of the strawberry leaves with sporulating colonies. Treated plants were then incubated under controlled conditions. Inoculated leaves were collected at 0, 24, 48, 72, 96, 120, 144, and 168 h post-inoculation (hpi). Drought stress treatment was performed by withholding water followed by sampling at 0, 24, 48, 96, 120, 144, and 168 h post-treatment (hpt). Plants were recovered after 168 h of drought stress by watering and sampled after an additional 24 h. Salt stress treatment was applied to 3-month-old substrate-grown strawberry seedlings via irrigation with 300 mM NaCl. Treated leaves were sampled at 0, 0.5, 2, 4, 8, 12, 24, and 48 hpt. Heat stress treatment was applied by transferring the seedlings to 42°C, and the treated leaves were collected at 0, 0.5, 2, 4, 8, 12, 24, and 48 hpt. For the hormone treatments, the strawberry leaves were sprayed with 0.1 mM ABA, 1 mM SA, 0.1 mM MeJA, or 0.5 g/L Eth, and the leaves were collected at 0, 0.5, 2, 4, 8, 12, 24, and 48 hpt for RNA isolation. All of the plants were treated in the light, and three independent experiments were performed.

### Semi-Quantitative Reverse-Transcription PCR

Total RNA was extracted from treated strawberry leaves and different tissues exposed to normal conditions using the E.Z.N.A. Plant RNA Kit (Omega, Guangzhou, China) according to the manufacturer’s instructions. First-strand cDNA was synthesized from 2 μg of total RNA using PrimeScript RTase (Takara, Dalian, China). The concentration of cDNA was adjusted according to the internal reference gene *Fv18S* (GenBank accession: XM_011464048). We also use two additional internal reference genes, *FvGAPDH1* (GenBank accession: XM_004306515) and *FvGAPDH2* (GenBank accession: XM_004309993) to evaluate the quality and consistence of the cDNA (Francisco et al., 2013). RT-PCR reactions were conducted using the following profile: initial denaturation at 94°C for 2 min, followed by 30–36 cycles of denaturation at 92°C for 30 s, annealing at 60 ± 3°C for 30 s, extension at 72°C for 30 s, and a final extension at 72°C for 2 min. PCR products were separated in a 1.5% (w/v) agarose gel stained with ethidium bromide and imaged under UV light for further gene expression analysis. Each reaction was repeated three times, and the three independent analyses revealed the same trends for each gene and treatment. Expression levels measured by semi-quantitative PCR were quantified as the fold-change in response to the experimental treatments relative to the control samples and are presented as heat maps that were generated using GeneSnap and Mev 4.8.1. The primers used for RT-PCR are listed in **Supplementary Table S1**.

### Plasmid Construction

The predicted full-length coding sequences of *FvCaMs* and *FvCMLs*, including *FvCaM2*, *CaM3* and *FvCML7, CML15*, and *CML28*, were amplified using high-fidelity Taq HS-mediated PCR from the cDNA of woodland strawberry *F. vesca* accession Heilongjiang-3 leaves. The amplified PCR products were cloned into the pBI221 vector (Clontech, Beijing, China) containing the *CaMV* 35S promoter and GFP using the ClonExpress II One Step Cloning Kit (Vazyme, USA), resulting in plasmids pFvCaMs-GFP and pFvCMLs-GFP. The elicitor protein gene, *INF1*, was cloned as previously reported (Kamoun et al., 1998). Plasmid pINF1-GFP was constructed as described above. The primers used for cloning genes and constructing vectors are shown in **Supplementary Table S1**.

### Protoplast Isolation, Transfection, and Treatment

Arabidopsis mesophyll protoplasts were isolated from 4-week-old Col-0 leaves using a previously described method (Yoo et al., 2007). The recombinant plasmids and control plasmid (pBI221) were isolated using the Omega Plasmid Mini Kit (Omega, Guangzhou, USA). The DNA concentration was adjusted to 500 ng⋅μL^-1^ per 5 kb of DNA. For transfection, 200 μL of *Arabidopsis* mesophyll protoplast was transferred into a 2-mL round-bottom microcentrifuge tube, in which 200 μL of protoplat solution was mixed with 10 μL of recombinant or control plasmid. The transfection procedure was performed as previously described (Yoo et al., 2007). After transfection, *Arabidopsis* protoplasts were incubated in the dark at room temperature for 16–18 h before examination by fluorescence microscopy. Images were acquired using an Olympus BX-51 inverted fluorescence microscope (Olympus, Japan). The imaged data were processed using Adobe Photoshop (Mountain View, CA, USA). For multiple treatments, *Arabidopsis* protoplasts were treated for a duration of 16–18 h with 50 μM H_2_O_2_, 100 μM SA, 100 μM MeJA or 20 μM Eth after transfection and then examined using fluorescence microscopy. For the 4 and 42°C treatments, *Arabidopsis* protoplasts were first incubated at room temperature for 12 hpt and then transferred into a 4 or 42°C chamber for another 4 h. All of the experiments were repeated three times.

### Transient Expression Assay in *N. benthamiana* Leaves

*Agrobacterium tumefaciens* strain GV3101 individual and GV3101 transformed with pBI221, pFvCaMs-GFP, pFvCMLs-GFP, or pINF1-GFP were grown overnight in LB medium supplemented with 50 mg/L of rifampicin, 100 mg/L of ampicillin and 25 mg/L of gentamicin. The *Agrobacterium* suspensions were centrifuged and resuspended in infiltration buffer (10 mM MgCl_2_, 10 mM MES, and 100 μM acetosyringone). The suspensions were adjusted to an OD_600_ of 0.5 and maintained at room temperature for more than 3 h. The bacterial suspensions were infiltrated into the leaves of 4-week-old *N. benthamiana* using a syringe without a needle. Plants infiltrated with *A. tumefaciens* were grown in a greenhouse at 23°C. For the transient assay of individual genes, we first injected each 100 μL of *Agrobacterium* suspension with pFvCaM-GFP, pFvCML-GFP, or pBI221 plasmid (as a control), and 24 h post-transformation, we injected 10 mM CaCl_2_, 10 mM EGTA or infiltration buffer solution (as a control) at the corresponding sites. The experiments were repeated three times with equivalent results. For the HR analysis, we first injected each 100 μL of *Agrobacterium* suspensions with pFvCaM-GFP, pFvCML-GFP, and pBI221 plasmid (as a control), and at 24 h post-transformation, we injected another 100 μL of each *Agrobacterium* suspension containing pINF1-GFP or GV3101 alone. The experiments were repeated three times with similar results.

## Results

### Characteristics of *CaM* and *CML* Genes in Strawberry (*Fragaria vesca*)

**FIGURE 1:**
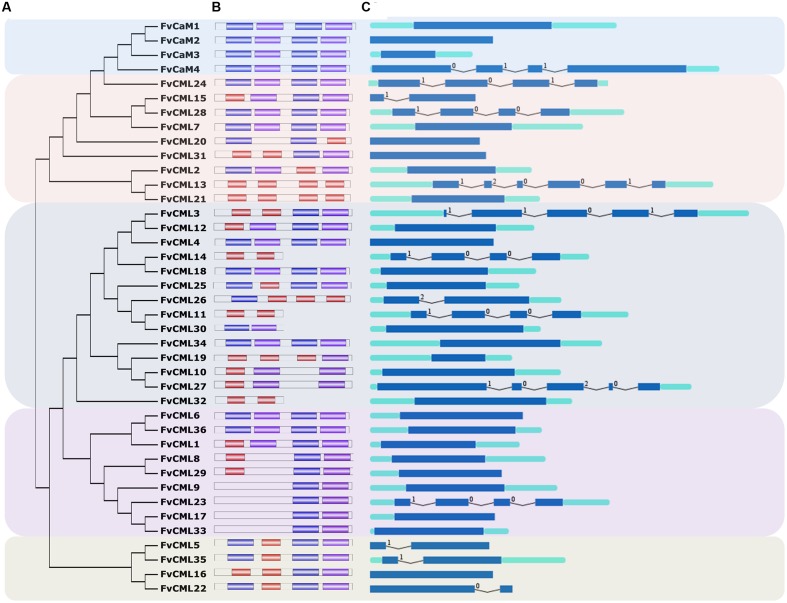
**Gene structure and phylogenetic relationship of *FvCaM* and *FvCML* genes.** **(A)** Phylogenetic tree of FvCaMs and FvCMLs. The phylogenetic tree was constructed using the full-length protein sequences of FvCaMs and CMLs by the neighbor-joining method with 1,000 bootstrap replicates. **(B)** Motif organization of the *FvCaM* and *FvCML* genes. The three colored boxes represent three distinct EF-hand motifs. **(C)** Exon–intron structures of the *FvCaM* and *FvCML* genes. The exons, introns and UTRs are represented by blue boxes, fold lines and green boxes, respectively. The intron phases are annotated by 0, 1, and 2. The sizes of the exons are proportional to their sequence lengths. The *FvCaMs* and *FvCMLs* can be divided into five subgroups, which are denoted as subgroups 1–5 from top to bottom.

**FIGURE 2:**
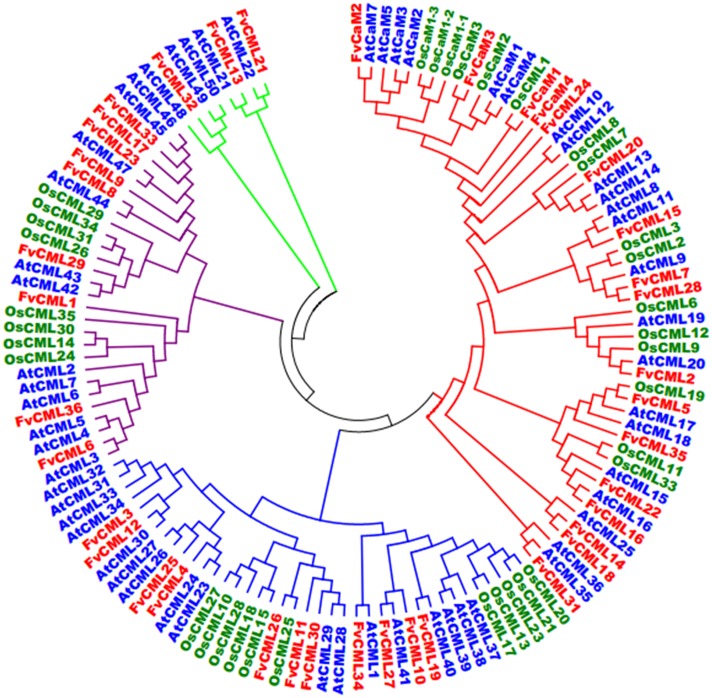
**Phylogenetic analysis of CaMs and CMLs in *Arabidopsis*, rice and strawberry.** The full-length amino acid sequences of CaMs and CMLs from *Arabidopsis* (At, blue), rice (Os, green) and strawberry (Fv, red) were aligned by ClustalX, and the phylogenetic tree was constructed by the neighbor-joining method with 1000 bootstrap replicates using MEGA 5.0.

To identify *CaM* and *CML* genes in the strawberry genome, we first determined the amino acid sequence of the *AtCaM1* gene of *Arabidopsis*. Next, we performed a Blast-P search against the strawberry genome (*F. vesca*) in NCBI using the amino acid sequence of AtCaM1 as the query. Protein sequences with an identity of more than 85% to AtCaM1 were defined as FvCaMs, and those with an identity of more than 16% were defined as FvCMLs. Preliminarily, we identified 8 *FvCaM* genes and 49 *FvCML* genes (data not shown). Further testing indicated that four (LOC101297640, LOC101308034, LOC101303028, and LOC101303236) of the eight *FvCaM* genes belonged to the *CML* group (**Figures 1A** and **2**), and some of the *FvCMLs* were recognized as CBLs and CDPKs. By removing the incomplete genes, transcripts of the same genes and redundant sequences, a total of 4 *FvCaM* genes and 36 *FvCML* genes were identified (**Table 1**). The putative protein sequences corresponding to the *CaM* and *CML* genes were submitted to Pfam and InterproScan to confirm the presence of the EF-hand domain and the absence of other functional domains. It is noteworthy that *FvCaM1*, which displayed a high level of homology to *OsCML1*, is still considered a *CaM* gene because it exhibits a high similarity (more than 90%) to *AtCaM1* and contains a typical CaM structure with four conserved EF-hands; a similar finding was obtained for *FvCaM4*. By contrast, *FvCML24* is closely related to CaMs phylogenetically, however, it lacks the typical EF-hand domain. Thus, we excluded it from the *CaM* group and defined it as a *CML* gene.

**Table 1:** Characterizations of *FvCaMs* and *FvCMLs.*

Name	Gene symbol^1^	Chromosome	Genomic location	Number of EF-hands^2^	Subgroup^3^
FvCaM1	LOC101310634	1	1453771..1454591	4	1
FvCaM2	LOC101306277	1	21925486..21927165	4	1
FvCaM3	LOC101308034	7	15905182..15905769	4	1
FvCaM4	LOC101308330	7	15907201..15908052	4	1
FvCML1	LOC101311505	1	2830239..2831418	4	4
FvCML2	LOC101295453	1	10976020..10977900	4	2
FvCML3	LOC101296230	1	11006585..11007694	4	3
FvCML4	LOC101299034	1	16734366..16743802	4	3
FvCML5	LOC101294531	2	5886641..5887270	4	5
FvCML6	LOC101294415	2	8519239..8520134	4	4
FvCML7	LOC101309501	2	16209859..16211260	4	2
FvCML8	LOC101311830	2	19404646..19405242	3	4
FvCML9	LOC101314049	2	22148679..22149342	2	4
FvCML10	LOC101313483	2	32740741..32741328	3	3
FvCML11	LOC101305440	3	5287246..5287736	2	3
FvCML12	LOC101296846	3	21600013..21604084	4	3
FvCML13	LOC101292979	3	22541413..22544316	4	2
FvCML14	LOC101302252	3	26376949..26386401	2	3
FvCML15	LOC101303028	4	5941675..5943796	4	2
FvCML16	LOC101313108	4	16571278..16572296	4	5
FvCML17	LOC101303326	4	17899738..17900262	2	4
FvCML18	LOC101304964	4	19239194..19239748	4	3
FvCML19	LOC101292012	4	20443557..20444330	4	3
FvCML20	LOC101299295	5	375772..376584	3	2
FvCML21	LOC101302652	5	4962858..4966366	4	2
FvCML22	LOC101297834	5	7131803..7132588	4	5
FvCML23	LOC101308877	5	8208587..8209177	2	4
FvCML24	LOC101297640	5	9793286..9795512	4	2
FvCML25	LOC101309155	5	10142011..10142805	4	3
FvCML26	LOC101295526	5	24687301..24688015	4	3
FvCML27	LOC101292606	5	26597114..26597991	3	3
FvCML28	LOC101303236	6	507656..509481	4	2
FvCML29	LOC101295534	6	6285677..6286493	3	4
FvCML30	LOC101307708	6	18470935..18471614	2	3
FvCML31	LOC101298441	6	23174861..23175773	4	2
FvCML32	LOC101306735	6	23523213..23525234	2	3
FvCML33	LOC101313141	6	31763246..31764212	2	4
FvCML34	LOC101296333	7	6711066..6711796	4	3
FvCML35	LOC101294800	7	20275502..20276323	4	5
FvCML36	LOC101310856	Un	165612..166327	4	4


*^1^Gene symbols are available in NCBI. ^2^The number of EF-hands was predicted using the MEME tool. ^3^These genes are grouped according to the phylogenetic tree.*

The putative 4 *FvCaM* genes and 36 *FvCML* genes were renamed based on their chromosome distributions. A series of correlative information for the *FvCaMs* and *FvCMLs* is shown in **Table 1**. The four *FvCaM* genes were distributed on chromosome Numbers 1 and 7, while the 36 *FvCML* genes were distributed across all seven chromosomes, excluding *FvCML36*, the location of which is still unknown in the strawberry genome. The *FvCaM* gene family contains four members, each of which is predicted to have four EF-hands. In contrast, the number of EF-hands in *FvCMLs* varies from two to four. The 4 *FvCaMs* and 36 *FvCMLs* were grouped into five subgroups, as shown in **Figure 1**.

### Phylogenetic Relationships of CaMs and CMLs among *Arabidopsis*, Rice and Strawberry

To investigate the evolution and to gain some insight into the potential functions of FvCaMs and FvCMLs, we constructed an unrooted phylogenetic tree using the full-length amino acid sequences from *Arabidopsis*, rice and strawberry. As shown in **Figure 2**, CaMs and CMLs were clearly separated but still displayed close phylogenetic relationships. FvCaMs clustered with AtCaMs and OsCaMs, with the one exception of OsCML1, which clustered with FvCaM1. In addition to CaMs, the CMLs clades were well defined. The CMLs from *Arabidopsis*, rice and strawberry clustered into four distinct clades, which were defined as subgroups 2 to 5 based on the orders. Subgroup 2 members displayed a high sequence similarity with CaMs and consisted of 13 FvCML members. Among them, FvCML24 showed the closest relationship with CaMs, supporting its potential function as a CaM. Subgroup 3 contained 11 FvCML members, most of which clustered with AtCMLs, excluding FvCML26, which showed a close phylogenetic relationship with OsCML25. In addition, nine FvCMLs were grouped in subgroup 4 and three in subgroup 5. Intriguingly, no OsCML was found in subgroup 5. Taken together, these findings showed that FvCaMs and FvCMLs clustered more frequently with *Arabidopsis* CaMs and CMLs than with rice.

### The Gene Structures of *FvCaMs* and *FvCMLs*

The gene structures were found to be relatively conserved among *FvCaMs* but variable among *FvCMLs*. The four *FvCaM* genes each contained the typical four EF-hands (**Figure 1B**), each of which had the ability to bind calcium ions. Motifs 1, 2, and 3 represent three distinct types of EF-hand (**Figure 1B**). *FvCaMs* only contained motifs 1 and 2, which compose a typical EF-hand domain pair. *FvCML* subgroup 2 members exhibited the closest phylogenetic relationship to *FvCaMs* and possessed a similar motif organization as *FvCaMs* (**Figure 1B**). In particular, the most closely related genes, including *FvCML24*, *CML28*, and *CML7*, were in the same mode as *FvCaMs* (**Figure 1B**). Subgroup 3 and 4 members exhibited a more complex motif organization. However, they still contained the similar motif organization, and some of the subgroups contained the same motif organization, such as *FvCML9*, *CML23*, *CML17*, and *CML33*, each of which carried one each of motif 1 and motif 2 (**Figure 1B**). Interestingly, subgroup 5 members had distant phylogenetic relationships from other *FvCMLs* (**Figure 1A**), but these four genes possessed conserved motif organizations. Regarding exon–intron structure, the majority of the *FvCaM* and *FvCML* genes lacked introns (**Figure 1C**). However, some of the genes contained intron insertions that did not follow any rule and that varied in amount and intron phase. Regardless, most of the phylogenetically closely related genes possessed the same or similar exon–intron structures. Taken together, the motif organizations and exon–intron structures revealed the conservation of gene structures. For example, *FvCaM1* and *CaM2*, *FvCML5*, and *CML35* possessed the same motifs and exon–intron organization (**Figures 1B,C**). Clustering of the motifs and exon–intron organization of the *FvCaM* and *FvCML* genes using an unrooted phylogenetic tree suggested an association between gene structures and evolutionary relationships.

### Synteny Analysis of *CaM* and *CML* Genes between *Arabidopsis* and Strawberry

**FIGURE 3:**
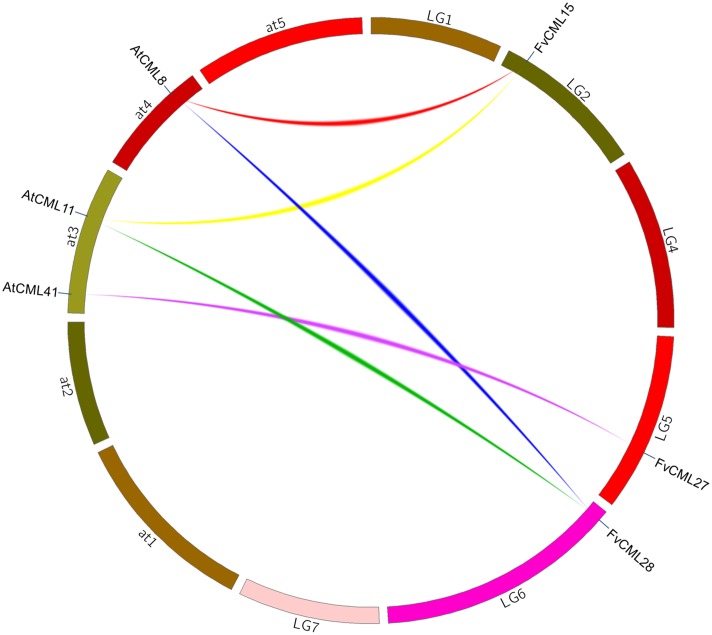
**Synteny analysis of *CML* genes from *Arabidopsis* and strawberry.** The chromosomes of *Arabidopsis thaliana* and strawberry are shown in different colors and in circular form. The approximate positions of the *AtCML* and *FvCML* genes are marked by a short blue line on the circle. Colored curves denote syntenic relationships between *Arabidopsis* and strawberry genes.

To further study the origin and evolution of *FvCaM* and *FvCML* genes, we investigated the synteny blocks between *Arabidopsis* and strawberry. In total, we found 24 pairs of syntenic genes containing *FvCaMs* or *FvCMLs* (data not shown). However, no syntenic relationship was observed between *FvCaMs* and *AtCaMs.* In addition, only 5 of the 24 pairs of syntenic genes were recognized as *FvCML-AtCML* gene pairs (**Figure 3**; **Supplementary Table S2**); and each pair of genes was phylogenetically closely related (**Figure 2**). It is worth noting that *AtCML8* exhibited two syntenic *CML* genes (*FvCML15* and *FvCML28*) in the strawberry genome, and *FvCML28* had two syntenic *CML* genes (*ATCML8* and *AtCML11*) in *Arabidopsis* (**Figure 3**). In addition, *FvCML15* was found to be phylogenetically closely related to both *AtCML8* and *AtCML11* (**Figure 2**). These relationships are quite complex, but we can still speculate that *AtCML8* and *AtCML11* are duplicated genes that arose before the divergence of *Arabidopsis* and strawberry. *FvCML28* and *FvCML15* were found to be duplicated genes that arose after the divergence of the two species. In addition, *FvCML27* and *AtCML41* were closely related in the phylogenetic tree (**Figure 2**), supporting the evolution of a single process. These results provide insight facilitating further investigations of the functions of *FvCML* genes.

### Multiple Alignment and Protein Structure of FvCaMs

**FIGURE 4:**
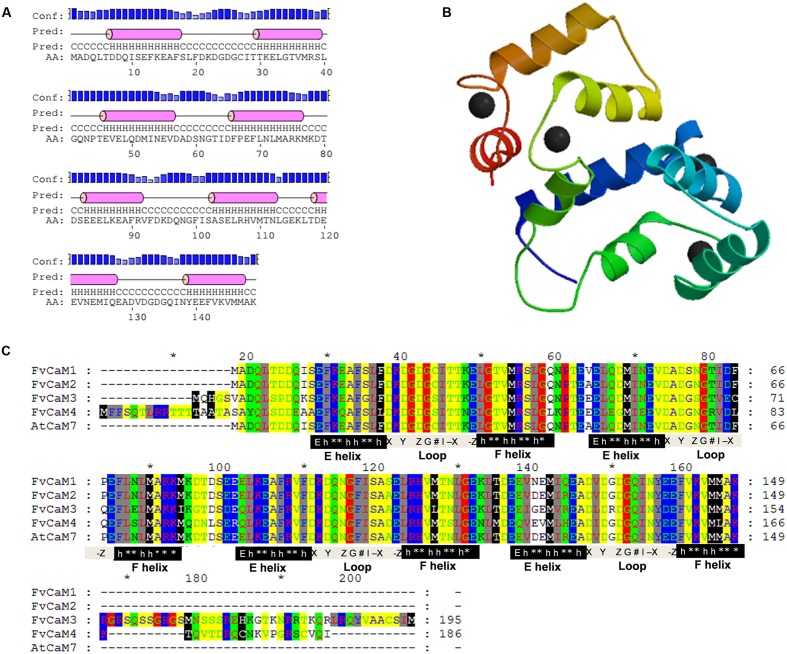
**Protein analysis of FvCaMs.** **(A)** Secondary structure of FvCaM1 protein. The purple cylinder indicates the helix in the EF-hand. **(B)** Three-dimensional structure of FvCaM1 protein. The 3D structure was predicted by SWISS-MODEL using AtCaM7 as template. Helix-loop-helix structures are shown in different colors, and the black spheres represent calcium ions. **(C)** The four FvCaMs were aligned with *Arabidopsis* AtCaM7. The aligned amino acids are shaded in different colors. The pivotal regions of the E helices, Ca^2+^-binding loops and F helices are represented by black, gray and black shades, respectively. The key sites for these regions are indicated beneath the relevant sequences. ‘E’ denotes glutamic acids, ‘h’ is hydrophobic amino acids, ‘^∗^’ indicates variable amino acids and the Ca^2+^-binding sites, X, Y, Z, G, #, -X, and -Z, are defined in the text.

Due to the similar sequences of CaMs among eukaryotic cells, the structures of CaMs are conserved. As shown in **Figure 4C**, the four FvCaMs aligned with *Arabidopsis* AtCaM7, the 3D structure of which has been reported (Tidow et al., 2012). The CaMs were composed of four EF-hand motifs, and each motif exhibited a helix-loop-helix structure (**Figure 4A**). FvCaM1 and FvCaM2 were extremely similar to AtCaM7, especially in terms of the EF-hand motifs. By contrast, FvCaM3 and FvCaM4, despite their variation in some amino acid sequences, were conserved at key sites in the EF-hand motif (**Figure 4C**). The canonical EF-hand motif of CaMs binds to Ca^2+^ via pentagonal bipyramidal geometry consisting of six coordination sites, with the sixth position located in the loop (Kawasaki et al., 1998). The corresponding six sites, the 1st, 3rd, 5th, 7th, 9th, and 12th amino acids (alternatively denoted X, Y, Z, #, -X, -Z) possess the ability to chelate Ca^2+^, as demonstrated by the 3D structure shown in **Figure 4B** (McCormack et al., 2005). The sixth position, which is usually a glycine (G), is thought to be important for the formation of the sharp turn within the loop. Notably, the seven sites involved in chelation are strongly conserved. The flanking E and F helices are generally composed of nine conserved amino acids with a regular distribution of hydrophobic amino acids (Kawasaki et al., 1998). By exploring the protein structures of FvCaMs, we gained a deeper understanding of the mechanism by which Ca^2+^ binds to CaMs, which will undoubtedly contribute to future research.

### Expression of *CaM* and *CML* Genes in Different Strawberry Tissues

Tissue specification of CaMs and CMLs is essential for protein interactions. In the present study, we tested the transcript abundance of the four *FvCaM* genes and four *FvCML* genes that are similar to *CaMs* in six different tissues (leaf, stem, root, bud, flower, and fruit) with the leaf examined in two stages (young and mature). As shown in **Supplementary Figure S3**, the eight genes were expressed in almost all of the tested tissues, yet some differences were still detected. For example, *FvCaM2* showed a high expression level in all of the tissues, while *FvCaM4* exhibited a steady low level of expression. In addition, *FvCaM1* was expressed at much higher levels in young than in old leaves. Notably, these eight genes were steadily and highly expressed in the bud, flower and fruit (**Supplementary Figure S3**), supporting their potential roles in plant reproductive growth and development processes. Exploring the expression of *FvCaM* and *FvCML* genes in different tissues will facilitate further investigations of their interacting proteins and their functions in calcium signaling.

### Expression Profile of *FvCaMs* and Several *FvCMLs* in Response to Multiple Treatments

To gain insights regarding the potential functions of *FvCaMs* and *FvCMLs*, we tested the transcription levels of the four *FvCaM* genes and the four most-related *FvCML* genes using RT-PCR following exposure to multiple treatments, including biotic and abiotic stresses as well as hormone treatments. Overall, *FvCaMs* and *FvCMLs* did not exhibit large changes in transcript abundance in response to the stress treatments; by contrast, some of them showed a distinct up-regulation in response to hormone treatment.

**FIGURE 5:**
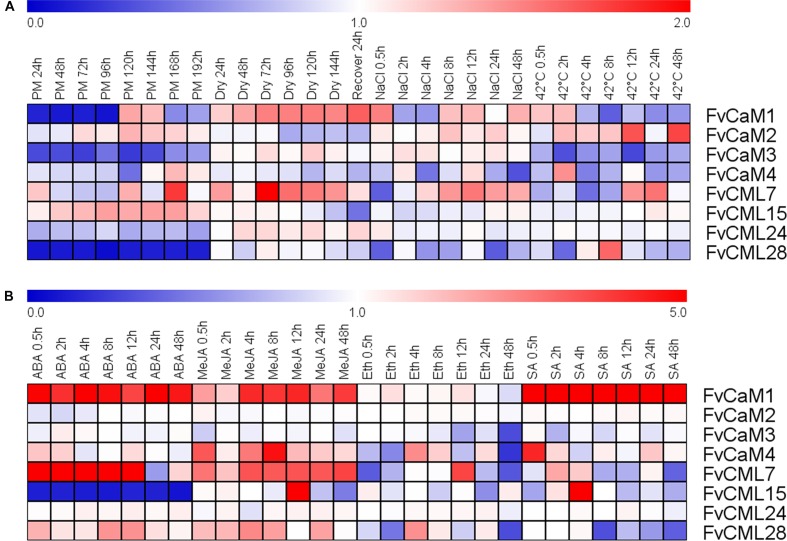
**Expression of four *CaM* and four *CML* genes in response to stress and hormone treatments in strawberry (*Fragaria vesca*).** Four stress treatments (powdery mildew, drought, NaCl and 42°C) **(A)** and four exogenous hormones (ABA, MeJA, Eth and SA) **(B)** were applied to the plants, and gene expression was measured by semi-quantitative reverse transcription PCR (RT-PCR). The *Fv18S* gene served as an internal control. The transcript abundance was measured and digitized using GeneSnap software and is indicated as the fold-change of the experimental treatments relative to the control samples visualized in a heat map. The color scale represents the log_2_ expression values, with red indicating increased transcript abundance and blue indicating decreased transcript abundance.

Strawberry PM was inoculated onto leaves as a biotic stress treatment, and drought, salt, and heat stress treatments were performed to simulate abiotic stress (**Figure 5**; **Supplementary Figure S1**). The *FvCaM* and *FvCML* genes exhibited insensitive expression patterns in response to multiple stimuli. However, some of these genes were clearly regulated. *FvCaM1* and *FvCML28* were strongly down-regulated (**Figure 5**), and the transcript abundance of *FvCaM1* immediately decreased at 24 hpi, returning to nearly normal levels at 120 hpi. In contrast, the transcript abundance of *FvCML28* decreased throughout the treatment period (**Figure 5**). In addition, *FvCaM3* was slightly down-regulated (**Figure 5**). Despite the distinct down-regulation patterns of the three genes discussed above, the responses of *FvCaMs* and *FvCMLs* to abiotic stress were more ambiguous. Only *FvCML7* showed a distinct change in expression at 72 hpt in response to drought treatment, with a subsequent return to normal expression levels after 24 h of watering (**Figure 5**). In addition, *FvCaM1* was slightly up-regulated in response to drought stress, even after the watering recovery period (**Figure 5**). For NaCl and the 42°C treatment, the expression patterns were more complex, and the expression levels fluctuated. It is worth mentioning that *FvCML15* and *FvCML24* were almost unresponsive to the three abiotic stress treatments (**Figure 5**), likely indicating that these two genes do not play a role in responses to abiotic stress.

The expression profiles in response to the hormone treatments revealed a distinct up- or down-regulation of the evaluated genes (**Figrue 5**; **Supplementary Figure S2**). Among the eight chosen genes, *FvCaM1* was remarkable because it was up-regulated throughout the entire treatment period in response to hormones, excluding Eth treatment, and it showed an especially strong up-regulation and reached peak levels of more than fivefold in response to SA treatment (**Figure 5**). Following ABA treatment, *FvCML7* also displayed an increase in transcript abundance from 0.5 to 12 hpt, returning to normal levels at 24 hpt (**Figure 5**). In contrast, *FvCML15* remained unexpressed during the whole treatment period (**Figure 5**). In addition to ABA treatment, several genes also responded to MeJA treatment. *FvCaM1*, *CaM4*, *CML7*, and *CML15* were positively regulated following MeJA treatment (**Figure 5**). Among them, *FvCaM1*, *CaM4*, and *CML7* showed an immediate up-regulation, but only *FvCaM4* exhibited an obvious peak at 8 hpt (**Figure 5**). In contrast, *FvCML15* was only up-regulated at 12 hpt and peaked at values of more than fivefold (**Figure 5**). The tested *FvCaMs* and *FvCMLs* that responded to Eth treatment did not follow any rules. The most dramatic gene was *FvCML7*, which was rapidly down-regulated at 0.5 hpt and then up-regulated, reaching a peak at 12 hpt (**Figure 5**). It is worth noting that after 24 h of treatment, some of the leaf margins became dry; at 48 hpt, some of the leaves are tending to be withered. This finding might explain why some *FvCaMs* and *FvCMLs* were suddenly down-regulated at 48 hpt. For SA treatment, in addition to the continuously high up-regulation of *FvCaM1*, it also positively regulated *FvCaM4* and *FvCML15* (**Figure 5**). The transcript abundance of the latter two genes suddenly increased at 0.5 and 4 hpt, respectively (**Figure 5**).

### Subcellular Localization of FvCaMs and FvCMLs

**FIGURE 6:**
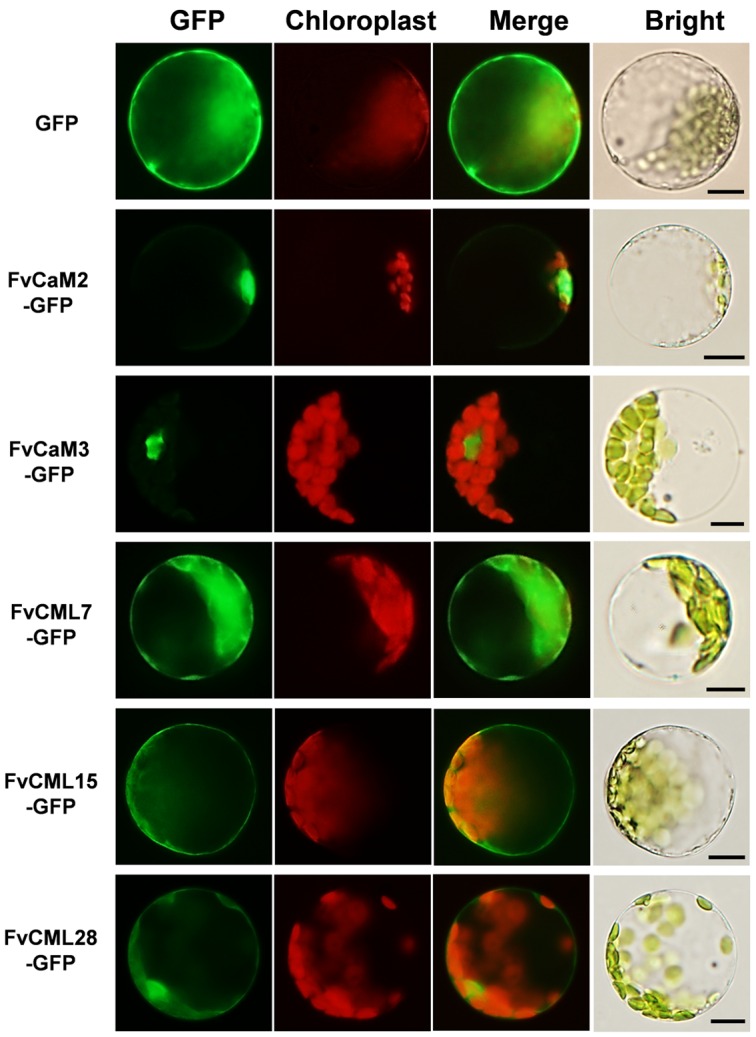
**Subcellular localization of five FvCaMs and CMLs.** The selected *CaM* and *CML* genes were cloned from woodland strawberry (*F. vesca*) and used to construct CaMV35S::CaMs–GFP and CaMV35S::CMLs–GFP vectors in which GFP was fused at the C terminus. The fusion proteins and GFP (as control) were transiently expressed in Col-0 *Arabidopsis* protoplasts and detected by fluorescence microscopy. The merged images include the green fluorescence channel (first panels) and the chloroplast auto-fluorescence channel (second panels). The corresponding bright field images are shown on the right. Bar = 10 μm.

The interactions of CaMs and CMLs with downstream proteins rely on the distributions and localizations in plant cells. To provide more cellular evidence, we cloned and isolated two *FvCaM* (*FvCaM2* and *FvCaM3*) genes and three *FvCML* (*FvCML7*, *FvCML15*, and *FvCML28*) genes to assess their subcellular localization. GFP served as a control. The fusion proteins were transformed into prepared *Arabidopsis* mesophyll cell protoplasts and observed using an Olympus fluorescence microscope. Our results revealed that FvCaM2 and FvCaM3 specifically localized in the nucleus, while FvCML7 and 28 were distributed throughout the whole cell, which was similar to the localization pattern observed for the GFP control (**Figure 6**). Another CML gene, FvCML15, was only detected in the plasma membrane and cytosol, but no specific nuclear localization signal was detected. Intriguingly, as the spearhead in the calcium signaling pathway, FvCaM2 and FvCaM3 did not display a plasma membrane localization pattern but were restricted to the nucleus (**Figure 6**), which might be due to downstream interacting proteins that are present in the nucleus. By contrast, the three FvCMLs were localized on the plasma membrane and throughout the cytosol; FvCML7 and FvCML28 were also localized in the nucleus (**Figure 6**), indicating their multiple roles in calcium signaling. These findings for FvCaMs and FvCMLs will facilitate further functional analyses of their targeted proteins.

**FIGURE 7:**
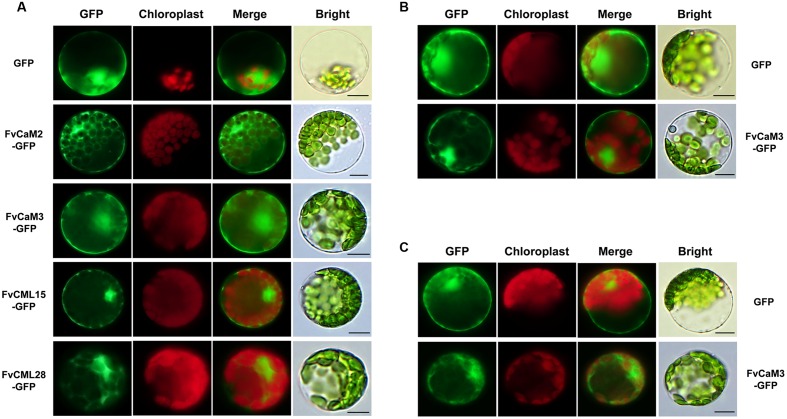
**Subcellular localization of several FvCaMs and FvCMLs in response to the treatments.** Three treatments, including MeJA **(A)**, Eth **(B)**, and 4°C **(C)**, were applied. Only the FvCaMs and FvCMLs that displayed changes in subcellular localization patterns in response to treatment are shown. **(A)** Subcellular localization of FvCaM2, CaM3, CML15, and CML28 following MeJA treatment. **(B)** Subcellular localization of FvCaM3 following Eth treatment. **(C)** Subcellular localization of FvCaM3 following the 4°C treatment. The experiments were repeated three times with consistent results. Bar = 10 μm.

In the present study, multiple treatments (4°C, 42°C, H_2_O_2_, SA, MeJA, and Eth) were applied to *Arabidopsis* protoplasts carrying pFvCaMs-GFP or pFvCMLs-GFP to explore changes in the subcellular localization of the two CaMs (FvCaM2, 3) and three CMLs (FvCML7, 15, 28). However, protoplasts treated with 42°C, H_2_O_2_ and SA were damaged and the fluorescence signal cannot be detected (data not shown). For the other three treatments (4°C, MeJA and Eth), the genes that showed a change in subcellular localization patterns are shown in **Figure 7**, while the genes that exhibited no changes in localization are not shown. Overall, FvCaMs and CMLs displayed a lower fluorescence intensity in response to the 4°C treatment, but the fluorescence intensity increased following treatment with hormones (MeJA and Eth). Under the 4°C treatment, a clear fluorescence signal for FvCaM3 was observed diffusely throughout the cytosol and nucleus, in contrast to the restricted localization to the nucleus under normal conditions (**Figure 7C**). In addition, following treatment with MeJA or Eth, FvCaM3 exhibited additional localization signal to the plasma membrane (**Figures 7A,B**). Only FvCaM3 showed clear changes in localization in response to the 4°C and Eth treatments; however, MeJA treatment resulted in changes in four genes (FvCaM2, CaM3, CML15, and CML28) (**Figure 7A**). The most intriguing one was FvCaM2, which exhibited a unique localization pattern. Compared with normal conditions, FvCaM2 displayed additional localization in the cytosol and, most likely, on chloroplast membranes (**Figure 7A**). Moreover, FvCML15 exhibited a distinct fluorescence signal in the nucleus, and the membrane localization signal of FvCML28 was lost (**Figure 7A**). Among the five tested genes, the localization of FvCaM3 changed in response to all of the evaluated treatments, while FvCML7 remained unchanged. Changes in the localization patterns of FvCaMs and CMLs will aid in elucidating their functions and interacting proteins in response to multiple treatments.

### Transient Expression Assays for *FvCaM* and *FvCML* Genes

**FIGURE 8:**
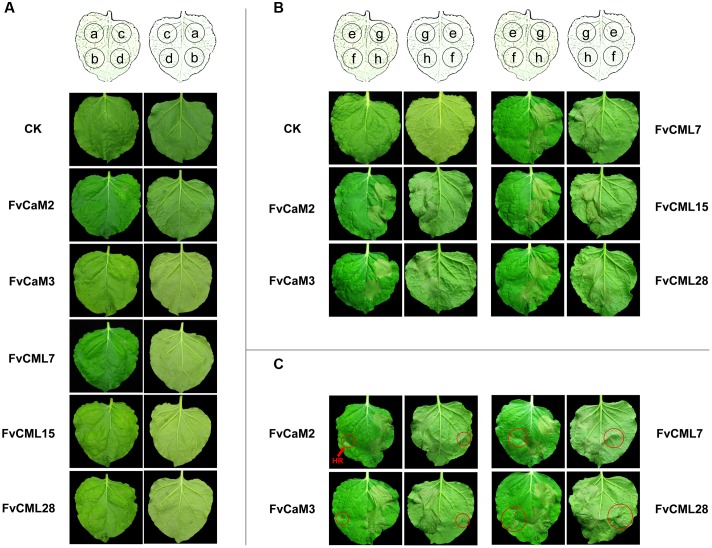
**Transient expression assays of FvCaMs and FvCMLs in the leaves of *Nicotiana benthamiana*.** **(A)** Transient expression of two FvCaMs and three FvCMLs that are unable to induce a hypersensitive response. Each leaf was infiltrated as shown in the schematic illustration in the front (left) and back (right) of the leaves. The leaves were infiltrated with *Agrobacterium tumefaciens* containing pFvCaM/CML-GFP at spot *a*, *b*, and *c*, followed by additional infiltration of CaCl_2_ (*b*) or EGTA (*c*). Spot *d* (as control) was infiltrated with the equivalent infiltration buffer. CK represents untreated *N. benthamiana* leaf. **(B,C)** Functional analysis of FvCaMs and FvCMLs in response to the pathogen-induced hypersensitive response. The leaves were infiltrated with *A. tumefaciens* containing pFvCaM/CML-GFP at spot *e* and *g*, or containing pBI221 (empty plasmid) at spot *f* and *h*. After a 24-h incubation, the leaves were infiltrated again with *A. tumefaciens* suspensions alone at spot *e* and *f*, or *A. tumefaciens* containing pINF1-GFP at spot *g* and *h. A. tumefaciens* infiltration sometimes induced a hypersensitive response (marked by red circles) **(C)** and sometimes resulted in no response, as observed at spot *f* **(B)**.

In this study, we transiently over-expressed *FvCaM* and *FvCML* genes in *N. benthamiana* leaves via *Agrobacterium*-mediated transfection. We also applied treatments with 10 mM CaCl_2_ and 10 mM EGTA as described previously (Reddy et al., 2011b) to provide the conditions of presence or absence of Ca^2+^. As shown in **Figure 8A**, these five genes did not induce HR or any other visible phenotypes following their transient over-expression in *N. benthamiana* leaves. In addition, these results were not changed by either CaCl_2_ or EGTA treatment. Because the tested *FvCaMs* and *FvCMLs* could not induce ROS burst, we further treated the infiltrated leaves with additional stimuli.

*CaM* and *CML* genes were found to participate in plant responses to biotic stress. Therefore, we conducted further experiments to evaluate the roles of *FvCaMs* and *FvCMLs* in the plant immune system. To avoid the systemic disease caused by pathogen inoculation, which might affect the control sites, we used an elicitor protein, INF1, which can induce the HR and cell death in *N. benthamiana* leaves, as previously reported (Kamoun et al., 1998). As shown in **Figures 8B,C**, the five *FvCaMs* and *FvCMLs* had no effect on the HR caused by INF1 (spot *g*), likely because these genes do not participate in pathways that are responsive to INF1. Intriguingly, we found that some of the spots that infiltrated by *A. tumefaciens* had HR phenotype (spot *f*, marked by red circles) (**Figure 8C**). In contrast, no HR was observed at the spots of over-expressed CaM/CMLs (spot *e*, infiltrated with GV3101 containing pFvCaM/CML-GFP and GV3101 alone, OD_600_ = 0.5) (**Figures 8B,C**). Among the five genes (*FvCaM2, CaM3, CML7, CML15*, and *CML28*), the infiltrated leaves of *FvCML15* did not show a HR response at spot *f*, while the other four genes displayed at least one transformed leaf with HR at spot *f*. In addition, the number of the infiltrated leaves that showed the HR phenotype at spot *f* differed among the four genes: *FvCaM2* (3/9), *FvCaM3* (3/9), *FvCML7* (7/9), *FvCML28* (2/9). These results indicated that *FvCaM* and *FvCML* contributed to the resistance of plants to *A. tumefaciens* and might participate in the immune response.

## Discussion

### Identification of *FvCaM* and *FvCML* Genes

Calmodulin, which functions as a Ca^2+^ sensor, is conserved among eukaryotic cells (DeFalco et al., 2010). In addition to the conserved CaMs, plants possess an extended family of CMLs. In the present study, we identified 4 *FvCaM* genes and 36 *FvCML* genes (**Table 1**). Due to a large number of genes encoding proteins that are similar to FvCaMs, we distinguished genes as FvCMLs based on sequence identities of more than 16%, as described for *Arabidopsis* (McCormack and Braam, 2003). Compared with the typical *CaM* genes (*FvCaM1* and *FvCaM2)*, *FvCaM3* and *FvCaM4* varied in gene length, especially *FvCaM4*, which was found to be much longer than typical *CaM* genes and which possessed three introns (**Figure 1C**). However, considering that *FvCaM3* and *FvCaM4* contained the typical EF-hand motif and clustered in the CaM group in the phylogenetic tree (**Figure 1**), we defined these two genes as *FvCaM* genes and considered them as *CaM* gene variants. Although it clustered with other *CaM* genes, *FvCML24* was not defined as a *CaM* gene due to the absence of the typical EF-hand motif (**Figure 1**).

### Bioinformatics Analysis of *FvCaM* and *FvCML* Genes Revealed Their Functional Conservation and Divergence

The conservation and divergence of gene structures leads to functional similarities or differences. Structural characteristics, such as the organization of motifs and exon–intron structures, provide insights concerning the evolution and function of *FvCaMs* and *FvCMLs*. All *CaMs* and *CMLs* are composed of EF-hands (McCormack et al., 2005). The number of EF-hands in *CaMs* and *CMLs* range from one to six (McCormack and Braam, 2003; Boonburapong and Buaboocha, 2007). However, only three EF-hands have been found in strawberry: two, three and four (**Table 1**). The EF-hand domain is conserved among *FvCaMs* (**Figure 4C**) but variable among *FvCMLs* (data not shown). Although *FvCaM3* and *FvCaM4* have different gene lengths and variable amino acid sequences, they still display conserved key sites, such as the EF-hand motif, and are considered to contain a typical EF-hand domain (**Figure 4**). The crystal structure of AtCaM7 has been described (Tidow et al., 2012). FvCaM1 has a sequence identity of 96.64% with AtCaM7, and we predicted the 3D structure of FvCaM1 protein using AtCaM7 as template (**Figure 4B**). These findings facilitated our exploration of the protein structure of FvCaM1 and the mechanism by which FvCaM1 binds to Ca^2+^. The great similarity of the sequences and protein structure between AtCaM7 and FvCaMs indicate their similar functions.

In eukaryotic cells, *CaM* gene have rarely varied during their evolutionary history, while *CMLs* are very diverse (DeFalco et al., 2010). Based on the phylogenetic tree (**Figure 2**), CaMs from *Arabidopsis*, rice and strawberry clustered within a group and were distinct from CMLs. FvCMLs more often clustered with AtCMLs than with OsCMLs, suggesting that CMLs are conserved among eudicots compared with monocots. Intriguingly, subgroup 5 contained five AtCMLs and three FvCMLs but no OsCML (**Figure 2**), probably because this cluster is only present in eudicots. Synteny analysis between *Arabidopsis* and strawberry provided additional information regarding the evolution of *FvCaMs* and *FvCMLs*. Intriguingly, *FvCaMs*, which are very conserved proteins evolutionarily, failed to be detected in synteny blocks with *Arabidopsis*. In contrast, five pairs of *CMLs* were recognized as syntenic gene pairs, and each pair of genes was found to be phylogenetically closely related (**Figures 2** and **3**). *AtCML8* and *AtCML11* possess two syntenic genes (*FvCML15* and *FvCML28*) in the strawberry genome, indicating that *FvCML15* and *FvCML28* arose prior to the divergence of *Arabidopsis* and strawberry. These syntenic genes are likely to have the same or similar functions (Ghiurcuta and Moret, 2014).

### The Expression Profiles and Subcellular Localizations of *FvCaM* and *FvCML* Genes Revealed Their Versatile Roles in Response to Multiple Stimuli

The roles of CaMs and CMLs during plant responses to stimuli and during plants growth and development have been widely examined (Reddy et al., 2011a). Based on previous microarray experiments, the expression patterns of individual members of the *CaM/CML* family have been shown to frequently differ spatially, temporally, and in magnitude in response to stimuli, suggesting specificity in their roles in signal transduction (Perochon et al., 2011). Our semi-quantitative RT-PCR data revealed an abundance of *FvCaM* and *FvCML* gene transcripts in response to multiple treatments and a spacial expression profile of tissues. Furthermore, stimuli can induce both spatial and temporal fluctuations in Ca^2+^ concentrations. As crucial mediators of calcium signaling, CaMs and CMLs respond to Ca^2+^ by interacting with numerous CBPs, which participate in the regulation of transcription, metabolism, ion transport, cytoskeleton-associated functions, protein phosphorylation, and phospholipid metabolism (Yang and Poovaiah, 2003; Bouche et al., 2005; Reddy et al., 2011a). These interactions rely on the specific locations in plant cells. As previously reported, the protoplast transient expression system is a powerful and versatile cell system for investigating gene expression in response to internal and external signals and for efficient and penetrating analysis of the underlying molecular mechanisms (Sheen, 2001; Yoo et al., 2007; Wu et al., 2014). A series of research used the *Arabidopsis* mesophyll protoplast transient expression system to study genes functions and signaling transduction (Kim and Somers, 2010; Zhang et al., 2015). Consequently, we used the *Arabidopsis* mesophyll protoplast isolation and transfection method to explore the subcellular localization of FvCaMs and FvCMLs and changes in their localization in response to treatments. The expression data together with the subcellular localization patterns provided evidence and contributed to facilitating the prediction of gene functions.

The significant roles of CaMs and CMLs in transducing pathogen-induced changes in Ca^2+^ concentrations to downstream proteins of the immune system have been well established (Chiasson et al., 2005; Boudsocq et al., 2010; Zhu et al., 2010). For example, *CaCaM1* (Choi et al., 2009) and *NtCaM13* (Takabatake et al., 2007), two genes that are induced in response to pathogens, have been shown to play negative roles via their transient over-expression and virus-induced gene silencing (VIGS), respectively. In addition to *CaMs*, *CMLs* such as *AtCML42* (Vadassery et al., 2012) and *AtCML43* (Chiasson et al., 2005) also enhance plant resistance to pathogens. In the present study, *FvCaM1*, *FvCaM3*, and *FvCML28* showed a decreased transcript abundance following inoculation with PM (**Figure 5**). The expression patterns were similar to those of two resistance-related *CaMs*, *CaCaM1* (Choi et al., 2009) and *NtCaM13* (Takabatake et al., 2007), which indicated that these three genes might be involved in immune pathways. Notably, the functions of some CaMs are associated with Ca^2+^ signaling and responses to SA, a hormone that is closely related to resistance. Through this pathway, CaMs were found to bind TFs (Du et al., 2009; Galon et al., 2010b). Similarly, *FvCaM1* was immediately and highly up-regulated in response to SA treatment (**Figure 5**). In summary, FvCaM1 is most likely to participate in immune signaling pathways (**Supplementary Figure S4**). In addition to *FvCaM1*, *FvCaM4*, and *FvCML15* were also positively regulated by SA treatment (**Figure 5**), and might also participate in the immune system. As discussed above, FvCaMs and CMLs function indirectly by interacting with proteins in immune signaling pathways, and additional proteins remain to be identified to fully understand the roles of CaMs and CMLs in the immune system.

In addition to biotic stress, CaMs and CMLs also participate in responses to abiotic stress, such as drought (Kaplan et al., 2006), salt (Xu et al., 2011), and heat (Zhang et al., 2009), as well as hormone treatments. TFs, such as bZIP and MYB, are considered to be the major CaM binding proteins in such pathways (Jakoby et al., 2002; Abe et al., 2003). The tolerance of plants to drought relies on ABA. CaMs and CMLs are recognized as important Ca^2+^ sensors in the ABA-mediated pathway (Kaplan et al., 2006). Our data showed that *FvCML7* had a distinct transcriptional response to drought stress, while *FvCaM1* had a slight response (**Figure 5**). This result is consistent with the expression pattern observed in response to ABA treatment, which resulted in the positive regulation of *FvCaM1* and *FvCML7* (**Figure 5**). Thus, these two genes tended to participate in the drought stress response (**Supplementary Figure S4**). In addition, a series of TFs are involved in the drought stress response, such as ABF/AREB (Fujita et al., 2005; Yoshida et al., 2010), MYB (Abe et al., 2003), and NAC (Rizhsky et al., 2004), and the binding of these TFs to CaMs occurs in the nucleus. Nevertheless, additional studies are needed to identify the specific TFs bound by FvCaMs and their corresponding signaling pathways. Compared with drought stress, a more subtle response was observed in strawberry following exposure to other abiotic stresses. In response to heat stress, the *CaM3* knockout *Arabidopsis* mutant displays impaired thermotolerance, whereas overexpression of *AtCaM3* increases thermotolerance (Zhang et al., 2009). The phylogenetically closely related *AtCaM3* gene in strawberry is *FvCaM2*, which also showed an increased transcript abundance in response to heat stress (**Figure 5**), indicating that it responds to heat stress similarly to *AtCaM3*. Additional findings revealed that CaMs participate in heat stress responses by interacting with HSFs (Liu et al., 2007), providing evidence that FvCaM2 is restricted to the nucleus (**Figure 6**). Intriguingly, *FvCaM2* only responded to the heat stress treatment and was insensitive to other stress and hormone treatments (**Figure 5**). These results emphasize the significance of *FvCaM2* responses to heat stress and the strong possibility that *FvCaM2* participates in heat response pathways (**Supplementary Figure S4**). In terms of NaCl stress, the responses of CaMs and CMLs have been published (Yoo et al., 2005; Galon et al., 2010a). A large number of CaM-binding proteins induced by salt stress have also been identified in the nucleus. For example, the CAMTA TF (Galon et al., 2010a) and CaMBP25 are nuclear CaM-binding proteins that are induced in response to multiple abiotic stresses (Perruc et al., 2004). In our study, *FvCaMs* and *FvCMLs* were insensitive to NaCl treatment (**Figure 5**), potentially due to the response to salt stress reflected in the activation of CaMs and CMLs proteins but not in their transcription levels. Notablely, for the treatments performed in *Arabidopsis* protoplasts herein, the most attractive gene was *FvCaM3*, which showed additional plasma membrane localization in response to the MeJA, Eth and 4°C treatments (**Figure 7**). These results might be due to its special function in Ca^2+^ signal transduction in response to treatment. However, more additional functional investigations are needed to confirm these hypothesis. One of the most important things for future research should be the identification of the physiologically relevant protein targets. Recent developments in protein interaction analysis, such as proteome chips, tandem affinity purification (TAP) and mass spectrometry could help to identify the downstream targets of CaMs and CMLs. And to further investigate the spatial and temporal distribution and the formation of protein complex *in vivo*, some cell biological approaches (e.g., bimolecular fluorescence complementation, fluorescence resonance energy transfer and other emerging technologies) could be used.

### Transient Expression Assays Suggested That Over-Expression of Four *FvCaM/CML* Genes Increased Resistance to *Agrobacterium tumefaciens*

*Agrobacterium*-mediated transient expression methods by leaf infiltration have been well-developed in *Nicotiana* species (Sheludko et al., 2007). As an efficient and versatile way, transient expression of exogenous genes in *N. benthamiana* leaves can provide preview of genes functions, especially for the HR phenotype (Goodin et al., 2008; Hao et al., 2014; Ouyang et al., 2014). A series of transient assays of *CaM* genes have been previously reported. Transient over-expression of *Capsicum annuum* calmodulin 1 (*CaCaM1*) activated the ROS burst and NO generation, and it induced HR-like cell death in pepper leaves (Choi et al., 2009). Silencing of *Solanum lycopersicum SICaM2* and *SICaM6* resulted in reduced resistance to Tobacco rattle virus and the oomycete pathogen, *Pythium aphanidermatum*, via VIGS (Zhao et al., 2013). Accordingly, we transiently expressed five *FvCaM* and *FvCML* genes in *N. benthamiana* leaves and exposed them to both the presence (Ca^2+^ treatment) and absence (EGTA treatment) of Ca^2+^. However, no significant HR phenotype was found (**Figure 8A**). Furthermore, we used an elicitor protein INF1 to evaluate whether over-expression of *FvCaM/CML* could increase or reduce H_2_O_2_ accumulation or the HR phenotype, yet no effects were observed (**Figures 8B,C**). However, we found that some sites with infiltrated *A. tumefaciens* containing pBI221 plasmid accumulated H_2_O_2_ and showed a slight HR phenotype (**Figure 8C**). In contrast, none of the sites that were infiltrated with *A. tumefaciens* containing pFvCaM/CML-GFP displayed HR phenotype (**Figure 8B**). As previously reported, plant resistance to *Agrobacterium* relies on the perception of PAMP, EF-Tu (Cyril et al., 2006). However, *N. benthamiana*, a plant that is unable to perceive EF-Tu, is highly sensitive to *Agrobacterium*-mediated transformation (Wroblewski et al., 2005). As previously supported, the HR and cell death induced by *A. tumefaciens* have been found in *N. benthamiana* and other species (Choi et al., 2009; Hao et al., 2014). Taken together, our results suggested that at least four *FvCaM/CML* genes (*FvCaM2, CaM3, CML7*, and *CML28*) could provide beneficial effects for plant resistance to *A. tumefaciens*. Because FvCaMs and FvCMLs were not responsible for INF1 and did not function as TFs or PRs (pathogenesis-related proteins), the observed resistance to *A. tumefaciens* might not be a result of their specific functions and might not be associated with a specific pathway. We postulate that the increased resistance of *N. benthamiana* leaves might be attributable to the following three factors: (i) Ca^2+^ signaling could be induced by stimuli (in this study, by our treatments); (ii) FvCaM/CML play roles upstream of Ca^2+^ signal transduction; (iii) over-expression of *FvCaM/CML* enhanced the perception and conduction of Ca^2+^ signaling, which improved the adaptation of the plants to the environments. Stable transformation experiments, including *Arabidopsis* mutants and transgenic strawberry plants, should be conducted to validate the functions of the *FvCaM/CML* genes.

## Conclusion

Calmodulins and CMLs act as bridge to connect Ca^2+^ signal and downstream CBPs and play vital roles in plants response to stimuli (Zhang and Lu, 2003; Lecourieux et al., 2006). During the past years, significant progress in our knowledge of the CaMs/CMLs in plants has been achieved (Perochon et al., 2011) in (i) identifying unique functions among members of this gene family, (ii) demonstrating the protein interactions between CaMs/CMLs and the targeted proteins, and (iii) describing the dynamic network that regulated by Ca^2+^. In this study, we identified 4 *CaM* and 36 *CML* genes in woodland strawberry (*F. vesca*). *FvCaMs* are conserved among eudicots, which compared with monocots. *FvCMLs* can be divided into four subgroups on the basis of phylogenetic relationship and gene structure. The transcript abundance of the tested *FvCaMs* and *FvCMLs* showed a distinct spacial and temporal regulation in different tissues and in response to multiple treatments. The comprehensive analysis combining the bioinformatics data and the experimental evidence indicated a series potential functions of FvCaMs and FvCMLs in biological process. The most intriguing genes and processes should be *FvCaM1* in biotic stress, *FvCaM2* in heat stress and *FvCaM1/7* in drought stress. However, a major challenge that remains is to further verify these predictions, determine their specificity, and test their biological significance. Overall, our study laid a foundation for further exploring the biological roles of CaMs and calmodulin-like proteins in strawberry plants.

## Author Contributions

KZ, JF, and ZZ conceived and designed the research. KZ carried out bioinformatics analyses. KZ, DY, and WW performed all treatments and RT-PCR tests. KZ and YH carried out subcellular localization experiments. KZ performed transient expression assays in *N. benthamiana* leaves. KZ and JF analyzed and interpreted the data. KZ draft the manuscript. ZZ and JF contributed with consultation. All authors read and approved the final manuscript.

## Conflict of Interest Statement

The authors declare that the research was conducted in the absence of any commercial or financial relationships that could be construed as a potential conflict of interest.

## Footnotes

1 http://www.ebi.ac.uk/interpro/search/sequence-search

2 http://pfam.xfam.org

3 http://www.Arabidopsis.org

4 http://rice.tigr.org

5 http://meme-suite.org

6 http://dog.biocuckoo.org/

7 http://gsds.cbi.pku.edu.cn/

8 http://chibba.agtec.uga.edu/duplication/

9 http://www.swissmodel.expasy.org
