# Supplementary material for: Characterization and Functional Analysis of Calmodulin and Calmodulin-Like Genes in Fragaria vesca
Source: Front Plant Sci. 2016 Dec 1;7:1820. doi: 10.3389/fpls.2016.01820 (PMC5130985; doi:10.3389/fpls.2016.01820)
Supplement: Supplementary file 1 [file Data_Sheet_1.docx]

**Supplementary materials**

The following are supplementary materials for this article:

**Supplemental Figure S1.** RT-PCR of *FvCaMs* and *CMLs* in response to stress treatments.

**Supplemental Figure S2.** RT-PCR of *FvCaMs* and *CMLs* in response to hormone treatments.

**Supplemental Figure S3.** Expression levels of *FvCaMs* and four *FvCMLs* in different tissues.

**Supplemental Figure S4.** Schematic diagram of the main findings and predicted functions of FvCaMs and FvCMLs.

**Supplemental Table S1**. Primers used in this study.

**Supplemental Table S2**. Synteny analysis of *FvCML* genes from *Arabidopsis* and strawberry.

**Supplemental Table S1**. Primers used in this study.

| \| Primer name \| Sequence (5'-3')1 \| \| Description2 \| \| \| --- \| --- \| --- \| --- \| --- \| \| Fv18S-qF \| ACCGTTGATTCGCACAATTGGTCATCG \| \| Strawberry 18S \| \| \| Fv18S-qR \| TACTGCGGGTCGGCAATCGGACG \| \| Strawberry 18S \| \| \| FvGAPDH1-qF \| GGCTTCTATCTCAACCGGCTCGTCTT \| \| GAPDH1 \| \| \| FvGAPDH1-qR \| CTTCCCACTGCTCCCTGATCTCTGATAC \| \| GAPDH1 \| \| \| FvGAPDH2-qF  FvGAPDH2-qR \| CCAAGTAAGGATGCCCCCATGTTCG  TTGGCAAGGGGAGCAAGACAGTTGG \| \| GAPDH2 \| \| \| GAPDH2 \| \| \| FvCaM1qF \| TCAAGGAAGCCTTCAGCCTCTT \| \| FvCaM1 (RT) \| \| \| FvCaM1qR \| TGGTCCCGTTACTATCAGCATCA \| \| FvCaM1 (RT) \| \| \| FvCaM2qF \| GCGATGGCTGCATTACTACCAA \| \| FvCaM2 (RT) \| \| \| FvCaM2qR \| CTCCTCCTCAGAATCAGTGTCCTT \| \| FvCaM2 (RT) \| \| \| FvCaM3qF \| ATGAGGAGATCGGTGAGATGGTT \| \| FvCaM3 (RT) \| \| \| FvCaM3qR \| CTGTTTAGTACGGCGGTTCTTAGT \| \| FvCaM3 (RT) \| \| \| FvCaM4qF \| GATGGATGAGCAGGTTGAGGTG \| \| FvCaM4 (RT) \| \| \| FvCaM4qR \| GCATTGTCTGTCAGTAACTTGTGTT \| \| FvCaM4 (RT) \| \| \| FvCML7qF \| GAACGACCGACGAAGGAAGAAG \| \| FvCML7 (RT) \| \| \| FvCML7qR \| CCTAGCCATGATACTCAAGAACTCA \| \| FvCML7 (RT) \| \| \| FvCML15qF \| CTACTATGTCCTCAATTCTTGGTTGC \| \| FvCML15 (RT) \| \| \| FvCML15qR \| ATGTCCTGAAGTTCCTCCTCTGT \| \| FvCML15 (RT) \| \| \| FvCML24qF \| AAGGATACCGATTCTGAGGAGGA \| \| FvCML24 (RT) \| \| \| FvCML24qR \| GCCATCACCATCTTCATCTGCT \| \| FvCML24 (RT) \| \| \| FvCML28qF \| TATTGACGGCAACGGAACCATA \| \| FvCML28 (RT) \| \| \| FvCML28qR \| CCATCTTGATCCTTATCGAACACTT \| \| FvCML28 (RT) \| \| \| FvCaM2-PBIF \| GAGAACACGGGGGACTCTAGAATGGCCGATCAGCTCACCGAC \| \| FvCaM2 (C, V) \| \| \| FvCaM2-PBIR \| ACCCATGGTACCCCGCTCGAGCTTGGCCATCATGACTTTGACG \| \| FvCaM2 (C, V) \| \| \| FvCaM3-PBIF \| GAGAACACGGGGGACTCTAGAATGCAACATGGTAGCGTGGC \| \| FvCaM3 (C, V) \| \| \| FvCaM3-PBIR \| ACCCATGGTACCCCGCTCGAGCATTATGCTACATGCAGCAACATATTG \| \| FvCaM3 (C, V) \| \| \| FvCML7-PBIF \| GAGAACACGGGGGACTCTAGAATGGCAGAAGCTTTAACAGAAACC \| \| FvCML7 (C, V) \| \| \| FvCML7-PBIR \| ACCCATGGTACCCCGCTCGAGGCTGAGCATCATCCTTGCAAATTC \| \| FvCML7 (C, V) \| \| \| FvCML15-PBIF \| GAGAACACGGGGGACTCTAGAATGGCAGAGGTACTAAGTGAAGAACAG \| \| FvCML15 (C, V) \| \| \| FvCML15-PBIR \| ACCCATGGTACCCCGCTCGAGTCCAATGGTCATCATCATCTTCAC \| \| FvCML15 (C, V) \| \| \| FvCML28-PBIF \| GAGAACACGGGGGACTCTAGAATGGGAGAGGTTCTAAGTGAAGAACAG \| \| FvCML28 (C, V) \| \| \| FvCML28-PBIR \| ACCCATGGTACCCCGCTCGAGGAAAGCTAATAACATCATTGTCACA \| \| FvCML28 (C, V) \| \| \|  \| \|  \| \|   1. Restriction sites are indicated in bold  2. The type of experiment for which the primers were used is indicated in brackets (RT: RT-PCR, C: cloning, V: vector construction). |
| --- | --- | --- | --- | --- | --- | --- | --- | --- | --- | --- | --- | --- | --- | --- | --- | --- | --- | --- | --- | --- | --- | --- | --- | --- | --- | --- | --- | --- | --- | --- | --- | --- | --- | --- | --- | --- | --- | --- | --- | --- | --- | --- | --- | --- | --- | --- | --- | --- | --- | --- | --- | --- | --- | --- | --- | --- | --- | --- | --- | --- | --- | --- | --- | --- | --- | --- | --- | --- | --- | --- | --- | --- | --- | --- | --- | --- | --- | --- | --- | --- | --- | --- | --- | --- | --- | --- | --- | --- | --- | --- | --- | --- | --- | --- | --- | --- | --- | --- | --- | --- | --- | --- | --- | --- | --- | --- | --- | --- | --- | --- | --- | --- | --- | --- | --- | --- | --- | --- | --- | --- | --- | --- | --- | --- | --- | --- | --- | --- | --- | --- | --- | --- | --- | --- | --- | --- | --- | --- | --- | --- | --- | --- | --- | --- | --- | --- | --- | --- | --- | --- | --- | --- | --- | --- | --- | --- | --- | --- | --- | --- | --- | --- | --- | --- | --- | --- |

**Supplemental Table S2**. Synteny analysis of *FvCML* genes from *Arabidopsis* and strawberry.

| **Genes from *Arabidopsis*** | | | **Genes from Strawberry** | | |
| --- | --- | --- | --- | --- | --- |
| **Chr.** | **Gene Locus** | **Name** | **Chr.** | **Gene ID** | **Name** |
| at3 | AT3G22930 | *AtCML11* | LG2 | gene29038 | *FvCML15* |
| at4 | AT4G14640 | *AtCML8* | LG2 | gene29038 | *FvCML15* |
| at4 | AT4G14640 | *AtCML8* | LG6 | gene16557 | *FvCML28* |
| at3 | AT3G22930 | *AtCML11* | LG6 | gene16557 | *FvCML28* |
| at3 | AT3G50770 | *AtCML41* | LG5 | gene29510 | *FvCML27* |
