# Supplementary figures and images for: Characterization and Functional Analysis of Calmodulin and Calmodulin-Like Genes in Fragaria vesca
Source: Front Plant Sci. 2016 Dec 1;7:1820. doi: 10.3389/fpls.2016.01820 (PMC5130985; doi:10.3389/fpls.2016.01820)

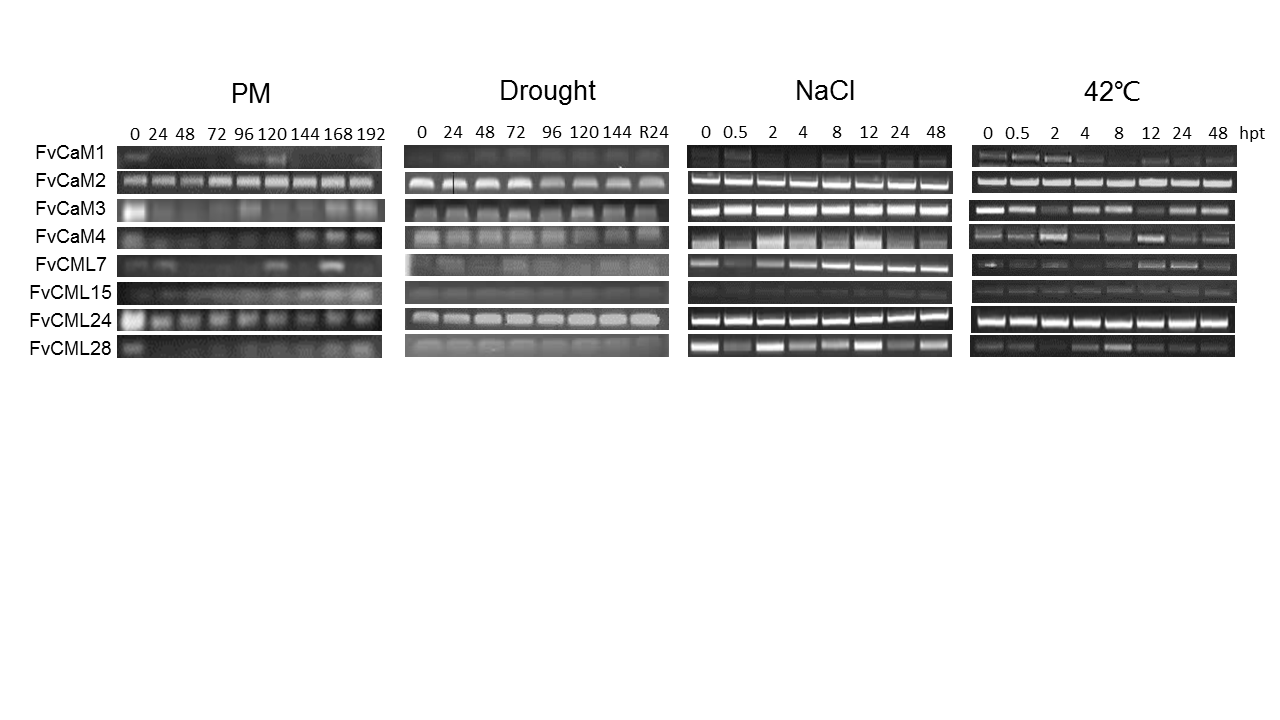

Supplement: Supplementary file 2 [file Image_1.TIF]

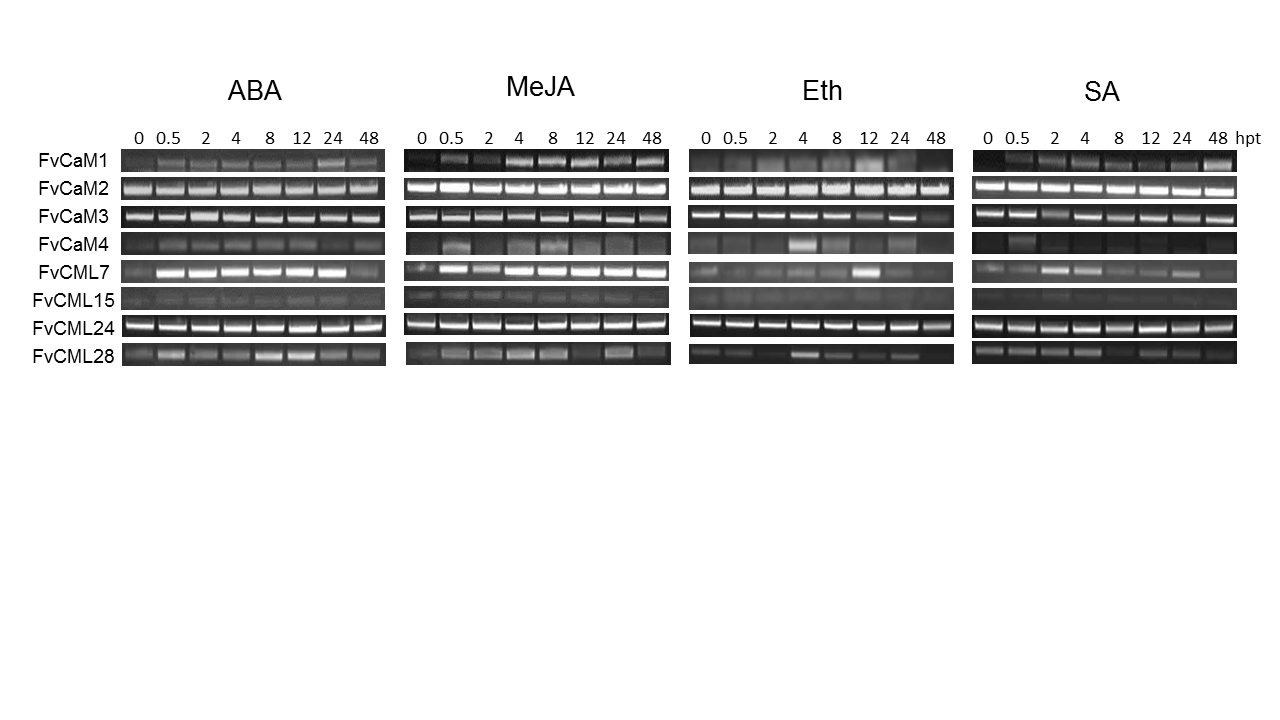

Supplement: Supplementary file 3 [file Image_2.TIF]

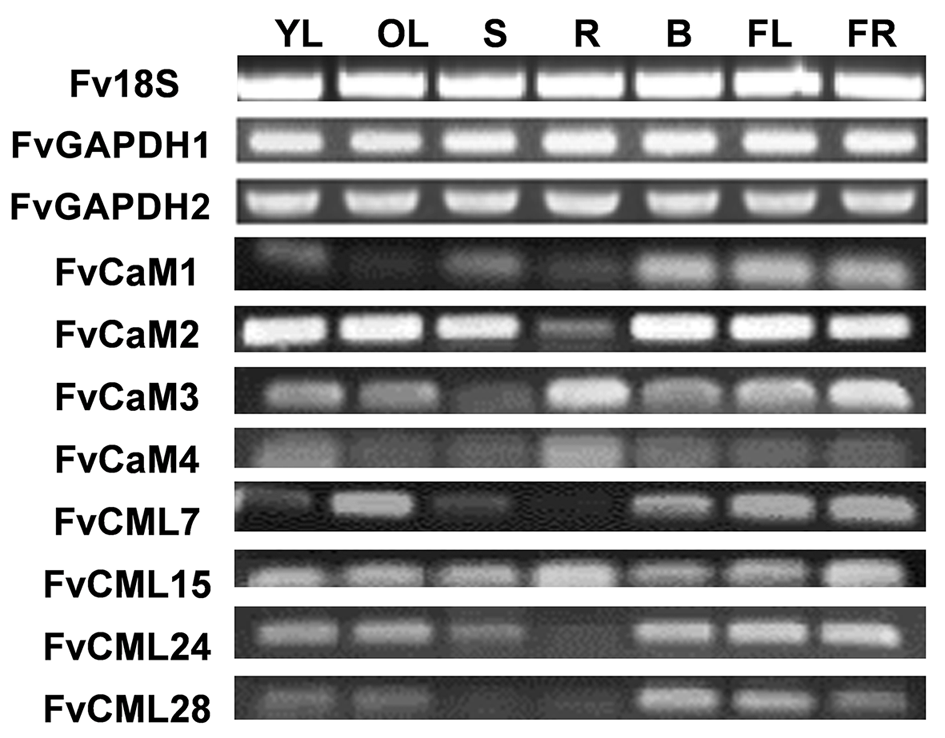

Supplement: Supplementary file 4 [file Image_3.TIF]

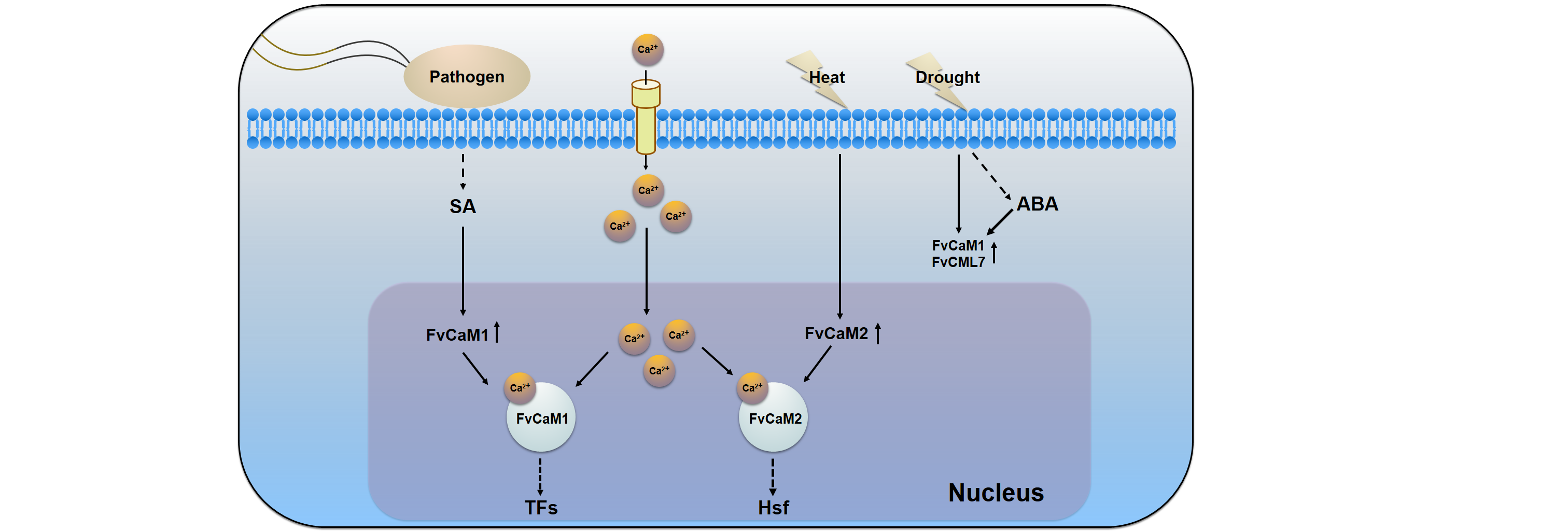

Supplement: Supplementary file 5 [file Image_4.TIF]
